# Polaritons light up future displays

**DOI:** 10.1038/s41377-024-01647-3

**Published:** 2024-11-04

**Authors:** Andreas Mischok

**Affiliations:** Humboldt Centre for Nano- and Biophotonics, Department of Chemistry, University of Cologne, Greinstr. 4-6, 50939 Köln, Germany

**Keywords:** Organic LEDs, Polaritons

## Abstract

Exciton-polaritons have long been a focus point of fundamental research towards polariton lasing, chemistry, and quantum optics. Recent developments now show their extraordinary potential for efficient and bright displays with ultimate color purity.

Organic light-emitting diodes (OLEDs) have revolutionized the display industry over the past 25 years and today, OLED displays can be found in a majority of high-end smartphones, as well as TVs, smartwatches, and many more consumer devices. While OLEDs offer high efficiency, facile fabrication, and chemical tunability, a major drawback is their emission linewidth, which is inherently broadened by the intrinsic disorder. To reach the color purity demanded by the BT.2020 recommendation however, RGB displays call for ultranarrow emission linewidths—to cover 98.4% (99.5%) of the BT.2020 color space requires linewidths better than 20 nm (10 nm)^1^ at optimized wavelengths over the full emission cone. There is now a considerable amount of research dedicated to developing materials that exhibit such narrow linewidths, among them organic multi-resonant thermally activated delayed fluorescence (MR-TADF) emitters^2,3^, colloidal quantum dots^4,5^, perovskites^6^ and inorganic quantum wells in micro-LED-displays^7^.

Apart from improving the light-emitting material, the color purity can also be enhanced by the use of a resonant microcavity, where the OLED structure is sandwiched between highly reflective surfaces with an approximate optical cavity thickness of *λ*/2 (or integer multiples). The resulting constructive and destructive interference can significantly narrow the emission spectrum around the design wavelength *λ*. This works even for intrinsically broad emitters, as the exciton decay into the resonant mode is enhanced by the amplified electromagnetic field through the Purcell effect^8^. As high-quality thin metal films such as silver can be simultaneously used as electrodes and cavity mirrors, this approach is highly desirable, in particular for the integration of top-emitting OLEDs on a complementary metal-oxide-semiconductor (CMOS) backplane for (micro-)displays. The use of microcavities however introduces a major drawback, as the resonance condition is dependent on the viewing angle of the device, leading to a significant blueshift at increasing angles. By increasing the light-matter interaction between excitons in the OLED structure and photons in the microcavity, this angular dispersion can now be remedied^9^. In the strong coupling regime, the coherent interaction between photons and excitons leads to a mixing of light and matter and in turn an energetic Rabi-splitting into upper and lower exciton-polariton (or simply polariton) quasiparticles. If the coupling parameters are tuned correctly, the resulting polariton dispersion can be close to the angle-independent excitonic dispersion. Polariton-based OLEDs have seen a significant increase in efficiency in recent years^9–12^ and can fulfill the requirements of narrowband and angle-independent emission. Going beyond current narrowband emitters, ultimate color purity can be achieved by a laser display, allowing for essentially monochromatic emission spectra.

In a recent publication in *Light: Science & Applications*, Jianbo De, Ruiyang Zhao, Fan Yin Hongbing Fu, Qing Liao et al. demonstrate high-brightness and color-pure polaritonic light-emitting diodes based on single organic crystals and showcase their potential towards realizing a polariton laser display^13^. By growing a high-quality single organic crystal of 1,4-dimethoxy-2,5-di(2,2’,5’,2”-terthiophenestyryl) benzene (TTPSB) inside a 150 nm Ag–35 nm Ag microcavity structure (see Fig. 1a, b of ref. ^13^), they demonstrate anisotropic strong coupling of multiple higher-order microcavity modes with the TTPSB excitons, resulting in the formation of two lower polariton branches with strongly reduced angular dispersion (Fig. 1c, d of ref. ^13^). The high optical quality of the organic crystal supported by the silver mirrors enables polariton lasing at an optical pump threshold of 15.8 μJ/cm^2^, accompanied by a nonlinear increase in intensity, a collapse of the PL decay time and a sudden narrowing of the emission linewidth down to 2 nm. Furthermore, De et al. demonstrate efficient charge carrier injection through the Ag contacts into the organic crystal by the addition of a hole injection layer of molybdenum trioxide and electron injection layers of calcium and cesium fluoride. The resulting OLED shows an ultranarrow emission spectrum originating from the second lower polariton mode (LP2) with a full width at half maximum (FWHM) of 4.1 nm at a central wavelength of 627 nm which is stable over an emission cone of 15°, very close to the BT.2020 recommendation for red emission (Fig. 3d of ref. ^13^). Even taking into account the remaining small angular dispersion of the lower polariton, the fully angle-integrated spectrum still shows a FWHM of less than 10 nm. In addition, due to the orientation of the organic crystal, the emission is intrinsically linearly polarized. While the driving voltage of the device is high due to the large thickness of the crystal, the polariton OLED remains exceptionally stable up to driving currents of 175 A/cm^2^ resulting in a luminance of 780,000 cd/m^2^. Future devices could be optimized for higher OLED performance by employing triplet harvesting, e.g. through the use of a TADF emitter in a crystalline host^14^. The driving voltage will need to be reduced in a display application for integration on a CMOS backplane, for example through the use of doped transport layers^15^.

The prospect of electrically driven polariton lasing in such an organic system is of particular interest and the current work presents a significant step towards this, showcasing low-threshold optically pumped polariton lasing and high-brightness OLED emission in the same device. To achieve the current injection of polariton lasing, losses from both the photonic as well as the excitonic part should be reduced. For the first, the current system could be embedded in a high-quality microcavity using distributed Bragg reflectors, which will significantly increase the photon lifetime in the system. To reduce excitonic losses, the non-radiative recombination of the material needs to be reduced with a resulting increase in its photoluminescence quantum efficiency.

Exciton-polaritons present a significant and as yet not fully explored playground for realizing narrowband light-emitting diodes in a multitude of material systems. The precise design of polaritonic cavities allows for harnessing the advantages of resonant cavity structures while minimizing their main drawback, the angular dependence. In addition, polariton lasing could reach a much lower threshold than photon lasing as no population inversion is required, bringing the prospect of a directly electrically pumped organic polariton laser close to reality.
